# CBL-interacting protein kinase 25 contributes to root meristem development

**DOI:** 10.1093/jxb/ery334

**Published:** 2018-09-18

**Authors:** Mukesh Kumar Meena, Niraj Kumar Vishwakarma, Vineeta Tripathi, Debasis Chattopadhyay

**Affiliations:** National Institute of Plant Genome Research, Aruna Asaf Ali Marg, New Delhi, India

**Keywords:** Arabidopsis, auxin, CIPK25, cytokinin, meristem, PIN, root, SHY2

## Abstract

Co-ordination of auxin and cytokinin activities determines root meristem size during post-embryonic development. Calcineurin B-like proteins (CBLs) and their interacting protein kinases (CIPKs) constitute signaling modules that relay calcium signals. Here we report that CIPK25 is involved in regulating the root meristem size. Arabidopsis plants lacking CIPK25 expression displayed a short root phenotype and a slower root growth rate with fewer meristem cells. This phenotype was rescued by restoration of CIPK25 expression. CIPK25 interacted with CBL4 and -5, and displayed strong gene expression in the flower and root, except in the cell proliferation domain in the root apical meristem. Its expression in the root was positively and negatively regulated by auxin and cytokinin, respectively. The *cipk25* T-DNA insertion line was compromised in auxin transport and auxin-responsive promoter activity. The *cipk25* mutant line showed altered expression of auxin efflux carriers (PIN1 and PIN2) and an Aux/IAA family gene *SHY2*. Decreased PIN1 and PIN2 expression in the *cipk25* mutant line was completely restored when combined with a SHY2 loss-of-function mutation, resulting in recovery of root growth. *SHY2* and *PIN1* expression was partially regulated by cytokinin even in the absence of CIPK25, suggesting a CIPK25-independent cytokinin signaling pathway(s). Our results revealed that CIPK25 plays an important role in the co-ordination of auxin and cytokinin signaling in root meristem development.

## Teaser

CIPK25, a calcium-regulated protein kinase, plays an important role in the co-ordination of auxin and cytokinin signaling in root meristem development.

## Introduction

Roots exhibit indeterminate growth which is regulated by external factors (Schenk and Jackson, 2002; Chapman *et al.*, 2012). Root cells are produced in the root apical meristem (RAM) region. The quiescent center (QC), a group of cells located at the rootward proximal border of the RAM, is considered as the organizing center. The cells that surround the QC constitute the stem cell niche (SCN) that gives rise to the different cell files of the root by cell proliferation. Each stem cell divides and the daughter cells undergo additional cycles of division and move longitudinally towards the shoot. Eventually, the proliferation rate of these cells decreases as they move upwards; at one point they show a very low probability of division and begin to elongate rapidly at the elongation zone (EZ). For practical convenience, the region between the QC and the first elongated cortex cell was considered as the RAM in several studies. A small domain of the RAM bordering the EZ shows a very low probability of cell division, but is yet to start cell elongation and is referred to as the transition domain (TD). The cells in this domain of the RAM grow at the same relative rate as the cells in the proliferative domain and pass to the EZ. As the rate of cell division is different for each cell type, the boundary of the TD towards the QC cannot be well demarcated (Verbelen *et al.*, 2006; Dello Ioio *et al.*, 2007, 2008; Perilli and Sabatini, 2010; Ivanov and Dubrovsky, 2013). Thus, a balance between cell division and differentiation is required for proper maintenance of meristem size.

Various phytohormones such as auxin, cytokinin, gibberellin, ethylene, and abscisic acid (ABA) play vital roles in maintenance of this balance (reviewed in Pacifici *et al.*, 2015). Auxin promotes cell proliferation in the RAM in various ways including long- and short-distance signals mediated by the polar auxin transporters Pin-Formed (PIN), essential for creating auxin gradients (Tanaka *et al.*, 2006). A high concentration of auxin promotes SCF^TIR1^-mediated degradation of the Aux/IAA family of transcriptional repressors that form heterodimeric complexes with auxin response factors (ARFs). Aux/IAA degradation releases ARFs from Aux/IAA-mediated repression, resulting in activation of auxin-responsive target genes (Kepinski and Leyser, 2005; Mockaitis and Estelle, 2008; Boer *et al.*, 2014). On the other hand, cytokinin drastically reduces growth of the RAM primarily by promoting cell differentiation (Beemster and Baskin, 2000; Dello Ioio *et al.*, 2008). Cytokinin signaling is mediated through a two-component system composed of membrane-localized Arabidopsis histidine kinases (AHKs) (Hwang and Sheen, 2001; Inoue *et al.*, 2001; To and Kieber, 2008). The signal is transmitted through phosphorelay to activate Arabidopsis response regulators (ARRs) that promote expression of cytokinin target genes (To *et al.*, 2004; To and Kieber, 2008). Transition from mitotic cells in the RAM to elongated cells in the EZ is accompanied by DNA polyploidization of cells (Dello Ioio *et al.*, 2007; Ishida *et al.*, 2010; Adachi *et al.*, 2011). Cytokinin activates expression of CCS52A1, which promotes DNA polyploidization through ARR2, and thereby regulates meristem size (Takahashi *et al.*, 2013). It is proposed that the position of the boundary between dividing and elongating zones is determined by the antagonistic interaction of auxin and cytokinin. Cytokinin-activated ARR1 directly activates expression of SHY2/IAA3, resulting in repression of ARF activity and down-regulation of auxin transporter PIN genes, causing suppression of cell division. On the other hand, a high concentration of auxin promotes degradation of SHY2 and releases ARFs from repression to promote PIN expression and cell division (Dello Ioio *et al.*, 2008;Mockaitis and Estelle, 2008). It has recently been shown that in addition to cytokinin-mediated modulation of auxin distribution by regulating PIN expression, cytokinin-dependent auxin degradation also plays an important role in positioning the TD by creating a region of auxin minima (Di Mambro *et al.*, 2017).

Calcium ions (Ca^2+^) plays a pivotal role in perception of external signals. An increase in free cytosolic Ca^2+^ ([Ca^2+^]_cyt_) is recognized by an array of Ca^2+^ sensors including calcineurin B-like proteins (CBLs). CBLs in concert with interacting kinases CIPKs (CBL-interacting protein kinases) relay the signal by phosphorylation of various substrates (Luan, 2009). Altered [Ca^2+^]_cyt_ homeostasis inhibits root cell elongation (Bai *et al.*, 2009; Wu *et al.*, 2017). The role of Ca^2+^ and calcium-regulated kinases in auxin signaling has been described in several reports (Thimann and Schneider, 1939; Fuglsang *et al.*, 2007; Duby *et al.*, 2009; Monshausen *et al.*, 2011; Takahashi *et al.*, 2012; Li *et al.*, 2016; Wang *et al.*, 2016; Santin *et al.*, 2017). In this study, we observed that Arabidopsis *CIPK25* T-DNA insertion lines exhibited a short root phenotype resulting from reduced RAM size. Restoring expression of *CIPK25* in one of these lines complemented the root phenotype, suggesting that this gene plays a role in root development. *CIPK25* expression was responsive to external application of auxin and cytokinin. A mutant lacking *CIPK25* expression displayed compromised auxin transport and low expression of PIN proteins. The short root phenotype of the *cipk25* line was restored in the SHY2 loss-of-function background. Our data suggest that CIPK25 plays a role in balancing auxin and cytokinin signaling in root development.

## Materials and methods

### Plant materials, growth conditions, and treatments

*Arabidopsis thaliana* wild-type (ecotype Columbia; Col-0) and mutant lines (SALK_079011 and SALK_029271) with T-DNA insertions in the exon of *AtCIPK25* (At5g25110) were procured from the Arabidopsis Biological Research Center. Homozygous lines with T-DNA insertions were screened using primers specific for T-DNA borders and the *CIPK25* gene. Both SALK_079011 and SALK_029271 lines were used for the initial phenotyping study. The remaining experiments were performed with SALK_079011 which was designated as the *cipk25* mutant. *P*_*PIN1*_*::PIN1-GFP*, *DR*_*5*_*::GUS*, and *shy2-24* lines were kindly provided by Dr Jason W. Reed (University of North Carolina, USA). *P*_*PIN2*_*::PIN2-GFP* and *arr1 arr12* lines were provided by Dr Ashverya Laxmi (National Institute of Plant Genome Research, India). The above-mentioned reporter lines were crossed with the *cipk25* mutant. F_3_/F_4_ homozygous plants were selected by genotyping and used in the experiments. For stable line generation, *35S::CIPK25*, *P*_*CIPK25*_*::GUS*, and *P*_*SHY2*_*::GUS* constructs were introduced into Col-0 plants by the *Agrobacterium*-mediated floral dip method as described before (Clough and Bent, 1998). The *P*_*CIPK25*_*::CIPK25* construct was introduced into *cipk25* mutant plants to generate the complementation line. T_3_ homozygous *P*_*SHY2*_*::GUS*-expressing plants were crossed with the *cipk25* mutant for further studies. All seeds were surface-sterilized, stratified, and sown on half-strength Murashige and Skoog medium (1/2 MS) plates supplemented with 1% sucrose and 0.8% agar. Arabidopsis plants were grown on an agropeat:vermiculite (3:1) mixture in growth chambers with 120 µmol µm^–2^ s^–1^ light under a 16 h light/8 h dark cycle at 21 ± 1 °C. For root phenotyping, all plants were grown vertically on 1/2 MS medium plates as mentioned above. Root length was marked on plates from 4 days post-germination (4 dpg) onwards. Images of 10-day-old seedlings were captured for all the plates; root length and per day growth rate were measured for individual seedling using ImageJ software (Abramoff *et al.*, 2004).

For analyzing fold expression of *CIPK25* in response to hormones, Col-0 seedlings were transferred to liquid 1/2 MS medium, 12 h prior to the treatment for acclimatization. Auxin [indole acetic acid (IAA)] at 5 µM and *t*-zeatin at 5 µM final concentration were added to liquid medium. Root tissues were collected from treated seedlings at different time intervals. For *CIPK25* expression studies at different developmental stages, root tissues were collected from seedlings grown on 1/2 MS plates. CIPK25 promoter activites were analyzed in *P*_*CIPK25*_*::GUS* lines in different tissues and at different developmental stages. Forty-five-day-old soil-grown plants were taken as matured plants. To determine promoter–reporter activity by staining, seedlings were grown on vertical 1/2 MS plates and treated with hormones for various periods before staining and microscopy.

### Expression analysis by real-time PCR

Plant tissues were ground to a fine powder with liquid N_2_, and total RNA was isolated using the TRIzol Reagent (Invitrogen) according to the manufacturer’s protocol. DNA-free total RNA (1 µg) was converted into single-stranded cDNA using oligo(dT_18_) primers and a High Fidelity cDNA synthesis kit (Roche Diagnostics GmbH, Germany). Gene-specific primers were designed using the NCBI primer design tool (http://www.ncbi.nlm.nih.gov/tools/primer-blast). For quantitative real-time PCR (qRT-PCR), primers were designed to produce 100–150 bp amplicons. qRT-PCR was done in optical 96-well plates on a Vii A 7 Real-Time PCR System (Applied Biosystems, CA, USA) using 2× Power SYBR Green PCR master mix (Applied Biosystems). A dissociation curve analysis was performed for all primer pairs, and all experimental samples yielded a single sharp peak at the amplicon’s melting temperature. *ACTIN2* (At3g18780) was used as internal control. Fold induction values of target genes were calculated with the ΔΔCT equation (Rao *et al.*, 2013) and are presented relative to the mRNA level of target genes in control conditions. Technical and biological replicates were used in all the assays. The primer pairs used are listed in Supplementary Table S1 at *JXB* online.

### Yeast two-hybrid assay

The CIPK25 full-length coding sequence (CDS) was fused to the GAL 4 DNA-binding domain in the pGBKT7-BD vector (Clontech Laboratories Inc.). The CDS of all CBLs (CBL1–CBL10) of Arabidopsis were cloned into the pGADT7-AD (Clontech Laboratories Inc.) to be expressed as a fusion with the GAL4 activation domain. The pGBKT7-BD and PGADT7-AD constructs were co-introduced into the Y2H Gold strain of yeast using the Matchmaker Gold yeast Two-Hybrid system kit (Clontech Laboratories Inc.). The yeast colonies harboring both the plasmids were selected on double drop-out (DDO) medium lacking leucine and tryptophan and were confirmed by colony PCR. Positive colonies selected on DDO plates were grown in liquid DDO media for 2 d, adjusted to an OD_600_ of 1, 0.1, and 0.01. A 10 µl aliquot of each dilution was spotted on quadruple drop-out medium (-Leu-Trp-Ade-His) with 2.5 mM 3-AT (3-amino-1,2,4-triazole). Plates were incubated at 30 °C for 5 d.

### Transient expression of fusion proteins and microscopic visualization

The CIPK25 CDS was cloned between the *Nco*I and *Spe*I sites of the pCAMBIA1305 vector to generate overexpression lines. To generate the *CIPK25* expression line under its native promoter, 2.6 kb of the promoter and coding region with the stop codon was amplified from genomic DNA and cloned in the *Sma*I site of the pBI101.2 promoter-less vector. For the promoter–β-glucuronidase (GUS) fusion construct, 2.6 kb fragments upstream of the translation start codon of the corresponding genes were amplified from genomic DNA and were cloned in the pBI101.2 vector at the *Bam*HI site (for the CIPK25 promoter) and *Sal*I/*Xba*I sites (SHY2 promoter). The *35S::CIPK25-YFP* construct generated by Gateway Technology (Invitrogen) in the pEG101 binary vector was introduced into *Agrobacterium tumefaciens* (GV3101) and infiltrated in *Nicotiana benthamiana* leaves for transient expression. For agro infiltration, overnight-grown cell cultures were collected and resuspended in 1 ml of infiltration buffer (10 mM MES-KOH, pH 5.6, 10 mM MgCl_2_, and 150 µM acetosyringone) to an optical density at 600 nm (OD_600_) of 0.6. The working suspensions were prepared by mixing the *Agrobacterium* culture harboring the *35S::CIPK25-YFP* construct with an organelle marker for the plasma membrane (PM-mCherry) (Nelson *et al.*, 2007) in a 1:1 ratio, incubated at room temperature for 3 h, and infiltrated onto the abaxial surface of fully developed leaves. Plants were cultivated for 2–3 d under growth room conditions. Subcellular fractionation of CIPK25–yellow fluorescent protein (YFP)-expressing plants was done by using the Qproteome Cell Compartment kit (Qiagen GmbH, Germany) by following the manufacturer’s instructions. Cytosolic, membrane, and nuclear protein fractions were separated by SDS–PAGE followed by western blot, and probed with anti-green fluorescent protein (GFP) (Abcam, UK) antibody. The blotted membrane was probed with the antibodies against fraction-specific marker proteins, for example anti-histone H3 (nuclear fraction) (Sigma-Aldrich, Saint Louis, MO, USA), anti-plasma membrane H^+^ ATPase (membrane fraction), and anti-cytosolic fructose-1,6-bisphosphatase antibodies (cytosolic fraction) (Agrisera, Sweden).

 For GUS reporter staining, Arabidopsis seedlings and different plant tissues were vacuum infiltrated with GUS staining solution [50 mM sodium phosphate buffer (pH 7.0), 2 mM EDTA, 0.12% Triton, 0.4 mM ferrocyanide, 0.4 mM ferricyanide, 1.0 mM 5-bromo-4-chloro-3-indolyl-β-d-glucuronide cyclohexylammonium salt] for 5–15 min and incubated in the dark at 37 °C. The staining time varied between 2 h and 12 h depending on the tissue type. Tissues were cleared of chlorophyll by treatment with 70% ethanol at 65 °C for 1 h, and analyzed by light microscopy.

For bimolecular fluorescence complementation (BiFC) assay, the full-length cDNA fragments (without the stop codon) of CBL4, CBL5, and CBL9 were ampliﬁed using PCR primers having attB adaptors and cloned into the Gateway entry vector pDONR 207 by BP clonase reaction (Invitrogen). The CIPK25 CDS was cloned into pSITE-nEYFP-C1 (CD3-1648) (Martin *et al.*, 2009) to generate the YFP^N^–CIPK25 construct. CBL4, CBL5, and CBL9 were cloned similarly into pSITE-cEYFP-N1 (CD3-1651) to generate CBL4–YFP^C^, CBL5–YFP^C^, and CBL9–YFP^C^ constructs. *Agrobacterium* strain GV3101 cultures harboring BiFC constructs were infiltrated in *N. benthamiana* leaves as discussed above. For fluorescence quantification, 6 mm leaf discs were cut out of the inﬁltrated areas and put into a black 96-well plate. Fluorescence intensities of leaf discs were measured in a The FLUOstar^®^ Omega plate reader (BMG LABTECH, Germany) using a 485 ± 12 nm excitation and a 520 nm emission ﬁlter. To examine root meristem cells, seedlings were incubated in 10 µg ml^–1^ propidium iodide for 5–10 min, mounted in water, and visualized by a Leica TCS SP5 (Leica Microsystems, Wetzlar, Germany) laser-scanning confocal microscope at 535 nm excitation and 617 nm emission for propidium iodide. GFP was visualized at 395 nm excitation and 509 nm emission in *P*_*PIN1*_*::PIN1-GFP*- and *P*_*PIN2*_*::PIN2-GFP*-expressing roots of Col-0, *cipk25*, and *CIPK25OX* seedlings. Agroinfiltrated *N. benthamiana* leaf cells for subcellular localization of CIPK25–YFP and plasma membrane marker fused with mCherry were analyzed at 514–527 nm and 587–610 nm for YFP and mCherry, respectively.

### Site-directed mutagenesis, bacterial expression, and kinase assay

Site-directed mutagenesis was done by replacing threonine (T) with aspartic acid (D) at the 201th position in CIPK25 as described (Meena *et al.*, 2015). The coding regions of CIPK25 and CIPK25T/D^201^ were cloned into the pGEX-4T2 vector (Amersham Pharmacia Biotech) at the *Sma*I site and transformed into *Escherichia coli* BL21 (DE3)-CodonPlus cells (Stratagene, La Jolla, CA, USA). Recombinant proteins were purified from the bacterial lysates by glutathione–Sepharose affinity chromatography as described before (Gong *et al.*, 2002a, b). *In vitro* protein autophosphorylation assays were performed in a 40 µl reaction mixture comprising purified recombinant glutathione S-transferase (GST) proteins incubated in kinase buffer (10 µCi of [γ-^32^P]ATP, 10 µM ATP, 20 mM Tris–HCl pH 8.0, 5 mM MnCl_2_, 1 mM CaCl_2_, 0.1 mM EDTA, and 1 mM DTT) for 30 min at 30 °C. For substrate phosphorylation, MBP (myelin basic protein) was incubated with immunoprecipitated CIPK25 protein from Col-0 seedlings in kinase buffer. The reaction was stopped by addition of 4× SDS sample buffer. Reaction samples were fractionated by electrophoresis and analyzed by autoradiography. CIPK25 protein was detected by a specific antibody generated against the CIPK25 immunogenic peptide.

### Auxin transport assay

Auxin transport was measured essentially as previously described (Shin *et al.*, 2005). Five-day-old vertically grown seedlings were used for the experiment. Briefly, agar blocks of 1 mm in diameter containing 7.7 × 10^−8^ M [^3^H]IAA (Amersham) were applied at different positions. Agar blocks were placed at the root tip for basipetal (shootward) and the hypocotyl–root junction for acropetal (rootward) auxin transport. After incubation for 3 h, a 0.5 mm section of the root close to the agar block was removed under the microscope. Two consecutive 2 mm segments below the incision line were then collected separately from 15 roots and were pooled and placed into glass scintillation vials containing 5 ml of scintillation fluid. The radioactivities in these two pools of root segments were measured using a Perkin Elmer Tri-Carb 2800TR Scintillation counter (Waltham, MA, USA).

### Statistical analysis

Statistical differences between different groups were detected by Student’s *t*-test and one-way ANOVA with post-hoc Student–Newman–Keuls (SNK) test in SigmaStat 2.03. Asterisks or different letters indicate significant differences between genotypes and treatments. Relative root growth rate in the presence of external IAA was given as the percentage growth rate (per day) of the same line in control conditions (without IAA).

## Results

### CIPK25 is involved in root meristem development

**Fig. 1.**
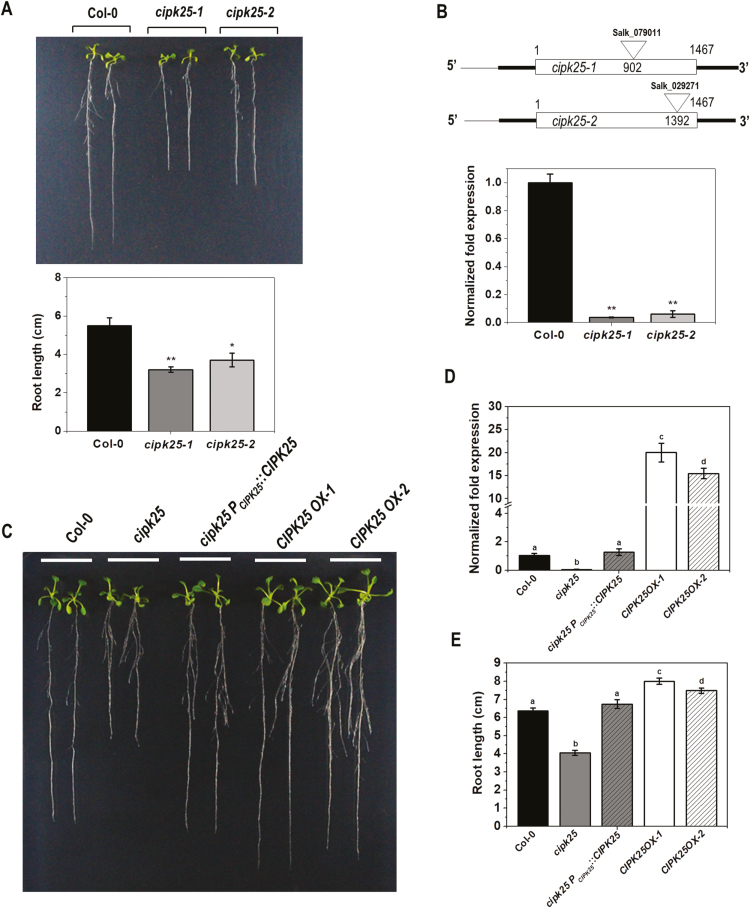
The *cipk25* mutant exhibits a shorter root length phenotype. (A) Primary root length phenotype of 8 dpg Col-0, *cipk25-1* and *cipk25-2* mutants grown vertically on 1/2 MS medium. The lower panel shows root length measurement of three replicate experiments with 15 seedlings for each replicate of each genotype. Error bars are ±SD. Statistically signiﬁcant differences between root lengths of Col-0 and *cipk25* mutants were analyzed by unpaired *t*-test: *P≤0.05, **P≤0.001. (B) Schematic representation of the *CIPK25* gene structure and T-DNA insertion sites of *cipk25-1* and *cipk25-2* mutants. The lower panel shows the relative transcript level of *CIPK25* in 8 dpg Col-0 and *cipk25* mutants by qRT-PCR. *ACTIN2* was used as the control. Error bars indicate ±SD of three replicates. Signiﬁcant differences were calculated by unpaired *t*-test: **P≤0.001. (C) Root length phenotype of 10 dpg Col-0, *cipk25*, *cipk25 P*_*CIPK25*_*::CIPK25*, *CIPK25OX-1*, and *CIPK25OX-2* lines. (D) *CIPK25* relative transcript levels in 10 dpg Col-0, *cipk25*, *cipk25 P*_*CIPK25*_::*CIPK25*, *CIPK25OX-1*, and *CIPK25OX-2* seedlings. *ACTIN2* was used as the endogenous control. Different letters indicate signiﬁcant differences (ANOVA; *P*≤0.05). (E) Root length comparison of 10 dpg Col-0, *cipk25*, *cipk25 P*_*CIPK25*_*::CIPK25*, *CIPK25OX-1*, and *CIPK25OX-2* seedlings. Error bars are ±SD, *n*=30.

We previously reported that overexpression of the chickpea *CIPK25* (*CaCIPK25*) in tobacco (*Nicotiana tabacum*) enhanced root growth (Meena *et al.*, 2015). Arabidopsis *CIPK25* (At5g25110.1) is an 1697 bp intron-less gene with a CDS of 1467 bp located between base pairs 8 657 626 and 8 659 322 on chromosome 5. Screening of T-DNA insertion lines (Alonso *et al.*, 2003) to investigate the role of Arabidopsis *CIPK25* identified two SALK lines, SALK_079011 (*cipk25-1*) and SALK_029271 (*cipk25-2*), with a T-DNA insertion in the *CIPK25* coding region having a root length shorter by ~40% and ~32%, respectively, as compared with wild-type (Col-0) plants at the 8 dpg stage (Fig. 1A). Sequencing of PCR products with T-DNA- and gene-specific primers identified the T-DNA insertions at 902 bp and 1392 bp downstream of the translation initiation site in *cipk25-1* and *cipk25-2*, respectively. Gene expression analyses by qRT-PCR showed that both mutant lines did not express a significant amount of *CIPK25* mRNA as compared with that in Col-0 plants (Fig. 1B). The SALK_079011 line was used for further study and designated as *cipk25*. Expression of the *CIPK25* full-length CDS under the 2.6 kb long native promoter in *cipk25* plants (*cipk25 P*_*CIPK25*_*::CIPK25*) functionally complemented the short root phenotype as observed in plants at 10 dpg. CIPK25-overexpressing [under the *Cauliflower mosaic virus* (CaMV) 35S promoter] Col-0 lines (*CIPK25OX*, line 1 and 2) displayed ~20% longer roots (Fig. 1C–E), suggesting that *CIPK25* is required for root development in Arabidopsis. *CIPK25OX* line 1 was used for further studies as it showed comparatively better expression and root growth than the other line.

**Fig. 2.**
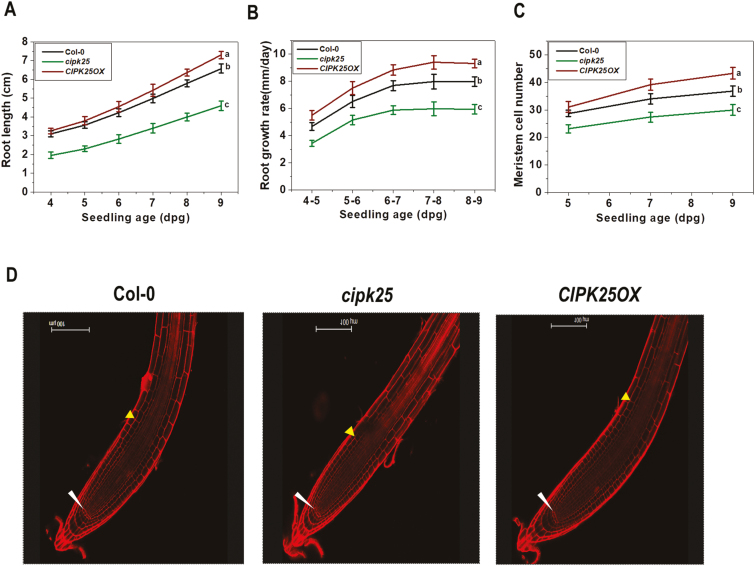
The *cipk25* mutant exhibits a slower root growth rate and a lower number of root meristem cells. (A and B) Primary root length and growth rate (mm d^–1^) measurements of Col-0, *cipk25*, and *CIPK25OX* seedlings grown vertically on 1/2 MS medium. Values are ±SD of 75 samples for each genotype. Different letters indicate signiﬁcant differences (ANOVA; *P*≤0.05) in seedlings at 9 and 8–9 dpg for root length and growth rate. (C) Meristem cell number count in Col-0, *cipk25*, and *CIPK25OX* in 5, 7, and 9 dpg seedlings. Error bars are ±SD of *n*=20. Different letters indicate statistically signiﬁcant differences among 9 dpg seedlings (ANOVA; *P*≤0.05). (D) Propidium iodide-stained primary roots of 5 dpg Col-0, *cipk25*, and *CIPK25OX* seedlings. Lower and upper arrows indicate the quiescent center (QC) and the ﬁrst elongated cortex cell, respectively.

Root lengths and growth rates of the Col-0, *cipk25*, and *CIPK25OX* plants were compared over a period of 4–9 dpg. No apparent differences were observed in germination rates and germination periods. Measurement of five replicates of 15 seedlings each showed that the average root length of the mutant line was shorter by 30–36% as compared with the Col-0 plants, whereas that of the *CIPK25OX* line was >11% longer at 9 dpg (Fig. 2A). Root growth rate, as measured by the increase in the primary root length per day, of the mutant line was ~25% less than that of the Col-0 plants when measured between the fourth and fifth day (Fig. 2B). During the same period, the root growth rate of the *CIPK25OX* line was similar to that of the Col-0 plant. Root growth rates of *cipk25* and *CIPK25OX* were ~26% less and ~17% more than that of the wild-type plants, respectively, when measured between the eighth and ninth day. To investigate the reason for the shorter root length in the mutant line, the number of root meristem cells was counted following the method of Perilli and Sabatini (2010). Propidium iodide-stained roots showed ~18–22% lower root meristem cell numbers in the *cipk25* line as compared with the wild type at 5–9 dpg. During the same period, the root meristem cell numbers for the *CIPK25OX* line were higher by ~10–20%. However, organization of cells in the root meristematic zone was apparently unperturbed in the mutant line (Fig. 2C, D).

### CIPK25 encodes an active protein kinase and interacts with CBL4 and CBL5

**Fig. 3.**
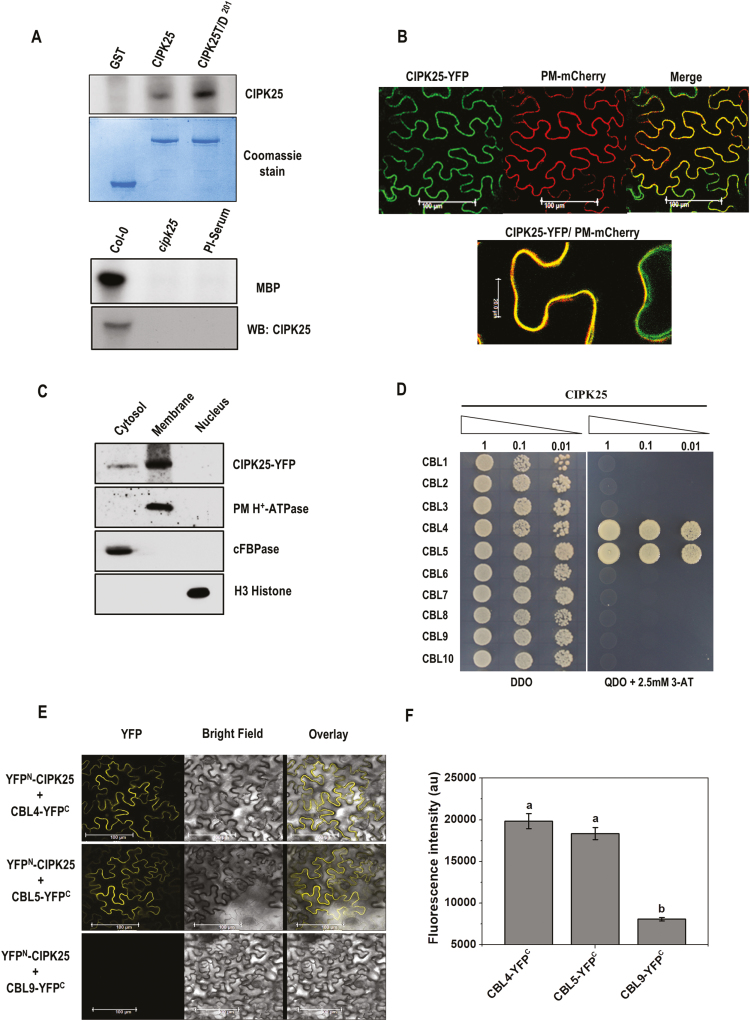
CIPK25, an active protein kinase, interacts with CBL4 and 5 *in planta*. (A) Autophosphorylation assay of bacterially expressed GST–CIPK25 and CIPK25T/D^201^ proteins. Below is the Coomassie blue-stained gel. The lower panel shows substrate phosphorylation of MBP by immunoprecipitated CIPK25 protein from Col-0 and *cipk25* mutant seedlings. Western blot of CIPK25 is shown. (B) Visualization of CIPK25–YFP fusion protein in agroinfiltrated leaves of *N. benthamiana* co-infiltrated with the plasma membrane marker PM-mCherry. The magnified view of the merged image in the lower panel shows CIPK25 localization at the plasma membrane and cytoplasm. (C) Western blot showing subcellular fractions of CIPK25–YFP-expressing *N. benthamiana* leaves. The blot was probed with antibodies against GFP for CIPK25, plasma membrane (PM) H^+^-ATPase, cytosolic fructose-1,6-bisphosphatase (cFBPase), and H3 histone. (D) Yeast two-hybrid interaction study of CIPK25 and CBLs. Y2H gold yeast cells were co-transformed with pGBKT7-CIPK25 and pGADT7-CBL1-10, and auxotrophic selection was carried out to detect protein–protein interaction. (E) Interactions between CIPK25 and CBL4/CBL5 *in planta* were shown by BiFC assay. CIPK25 fused with the N-terminal domain of YFP (YFP^N^) and CBL4/CBL5/CBL9 fused with the C-terminal domain of YFP (YFP^C^) were transiently expressed in *N. benthamiana* by agroinfiltration. Images show YFP-mediated fluorescence derived from the protein–protein interaction. Scale bar=100 µm. Infiltration of CBL9–YFP^C^ with YFP^N^–CIPK25 was used as the negative control. Expression of the proteins is shown in Supplementary Fig. S1. (F) Fluorescence intensity within leaf discs expressing YFP^N^–CIPK25 with CBL4–YFP^C^, CBL5–YFP^C^, or CBL9–YFP^C^. Different letters denote signiﬁcant differences among samples based on Student–Newman–Keuls test (*n*=8, ANOVA; *P*≤0.05).

To investigate its biochemical character, CIPK25 protein fused with GST was expressed in bacteria. GST–CIPK25 protein showed autophosphorylation activity in an *in vitro* reaction. Replacement of a conserved threonine residue with aspartic acid in the activation loop has been shown to increase autophosphorylation activity for various CIPKs (Gong *et al.*, 2002a, b). Similarly, substitution of the conserved Thr201 residue with aspartic acid resulted in an ~3-fold enhancement in the autophosphorylation activity of CIPK25. CIPK25 protein immunoprecipitated from Col-0 seedling protein extract showed *in vitro* kinase activity with MBP as substrate, implying that the plant CIPK25 protein is an active kinase (Fig. 3A). To explore the subcellular localization of CIPK25 protein, the CIPK25–YFP construct was agroinfiltrated in *N*. *benthamiana* leaves along with the plasma membrane marker PM-mCherry (Nelson *et al.*, 2007). Fluorescence overlay showed that CIPK25 was localized in both the plasma membrane and the cytosol (Fig. 3B). To substantiate the localization of CIPK25, subcellular fractionation of transiently expressed CIPK25–YFP in *N*. *benthamiana* leaves was performed. The presence of CIPK25 was detected by western blot using anti-GFP antibody mostly in the membrane and much less in cytosolic fractions, but not in the nuclear fraction (Fig. 3C).

Multiple CBL proteins can interact with a specific CIPK depending on context, and this is thought to define distinct signaling pathways (Kolukisaoglu *et al.*, 2004). A yeast two-hybrid assay was conducted to identify the interacting CBL partners of CIPK25. The full-length CIPK25 protein was found to interact only with CBL4 and CBL5 (Fig. 3D). *In planta* interaction of CIPK25 with CBL4 and CBL5 was investigated by BiFC assay in *N*. *benthamiana* leaves. Fluorescence due to reconstitution of YFP was observed when combinations of YFP^N^–CIPK25 with CBL4–YFP^C^ or CBL5–YFP^C^ were infiltrated in *N*. *benthamiana* leaves, whereas the combinations of YFP^N^–CIPK25 and CBL9–YFP^C^ did not show any fluorescence (Fig. 3E). Expression of the fused protein in the infiltrated leaf was verified by western blot (see Supplementary Fig. S1). To assess the strength of BiFC signals from YFP^N^–CIPK25/CBL4–YFP^C^ and YFP^N^–CIPK25/CBL5–YFP^C^ pairs, the fluorescence signal intensity of the leaf discs infiltrated with the BiFC constructs were quantified using a spectrofluorometer. No signiﬁcant differences were observed between the BiFC ﬂuorescence signals of YFP^N^–CIPK25 with CBL4–YFP^C^ and CBL5–YFP^C^, while the signals fromYFP^N^–CIPK25 and CBL9–YFP^C^ were negligibly low (Fig. 3F).

### Tissue-specific expression of *CIPK25*

**Fig. 4.**
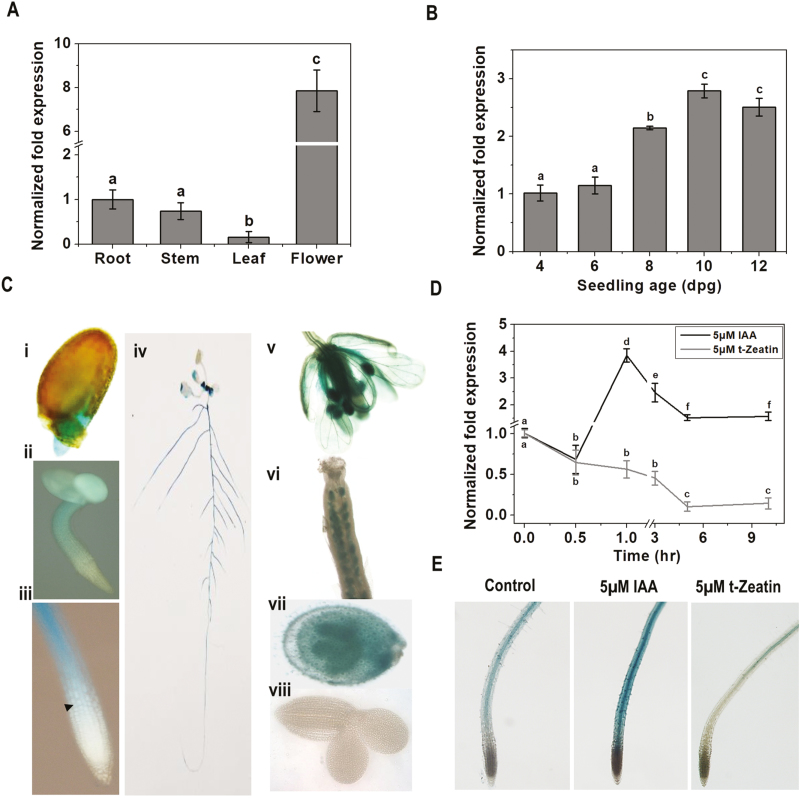
*CIPK25* gene expression profiles in different tissues, root growth stages, and in response to hormone treatments. CIPK25 gene expression in different tissues (A) and in different root growth stages (B) analyzed by qRT-PCR. *ACTIN2* expression was used for normalization. Error bars show ±SD, *n*=3. Different letters indicate signiﬁcant differences among samples (ANOVA; *P*≤0.05). (C) CIPK25 promoter activities were investigated with promoter::reporter gene (GUS) constructs expressed in transgenic plants. Histochemical GUS stainings is shown for (i) the radicle of a germinating seedling; (ii) a 2 dpg seedling; (iii) the primary root tip of a 5 dpg seedling (the arrow indicates the first elongated cortex cell); (iv) a 10 dpg seedling; (v) ﬂower; (vi) gynoecium; (vii) immature seeds with embryo; (viii) embryo. (D) Fold change in *CIPK25* expression as analyzed by qRT-PCR in roots of 10 dpg Col-0 seedlings treated with 5 µM IAA and 5 µM *t*-zeatin for different time intervals. *ACTIN2* expression was used for normalization. Error bars show ±SD, *n*=3. Different letters indicate signiﬁcant differences among samples (ANOVA; *P*≤0.05). (E) *pCIPK25::GUS* expression was visualized in primary roots of 10 dpg seedlings treated with 5 µM IAA for 1 h and with 5 µM *t*-zeatin for 5 h.

Expression of *CIPK25* was investigated in different tissues and root growth stages, and in response to auxin and cytokinin treatments by qRT-PCR. Expression of *CIPK25* was the lowest in leaves of the mature plant and highest in flowers, followed by the root and stem (Fig. 4A). Expression of *CIPK25* in the root increased gradually from the fourth to the 10th day by ~3 fold, and decreased to ~2.5 fold on the 12th day (Fig. 4B). To investigate the *in planta* expression pattern, the *GUS* reporter gene was cloned under a 2.6 kb 5' upstream regulatory region (including the 5'-untranslated region) of *CIPK25* (*P*_*CIPK25*_) and expressed in Arabidopsis. GUS activity could be detected throughout the radicles in the germinated seed. GUS activity was observed in cotyledons and hypocotyl, except in the proliferative region of the RAM in a 2-day-old seedling. In a 5-day-old seedling, GUS expression initiated at the TD and continued to the root–shoot junction. GUS activity in the aerial part of a 10-day-old seedling was not well pronounced where the blades of young leaves showed low levels of *GUS* expression. GUS activity progressively increased with age in the primary and lateral roots, except in the root tip. *P*_*CIPK25*_*::GUS* showed strong expression in the floral organs in adult plants. A high level of *CIPK25* expression was detected in carpel, petals, and stamens. The ovule was dissected and a strong *CIPK25* promoter activity was observed in ovule integuments, but not in the embryo (Fig. 4C). The plant growth hormone auxin (IAA) positively regulated *CIPK25* expression in root. The transcript level was increased by ~4-fold within 1 h of treatment with 5 µM IAA and then slowly decreased to ~1.8-fold within 5 h and remained at a similar level till 10 h. In contrast, cytokinin (5 µM *t*-zeatin) treatment down-regulated *CIPK25* gene expression by almost 10-fold within 5 h (Fig. 4D). Activation and repression of the CIPK25 promoter by auxin and cytokinin treatments was also validated by promoter–reporter assay (Fig. 4E).

### The *cipk25* mutant showed compromised auxin transport and auxin-responsive promoter activity

**Fig. 5.**
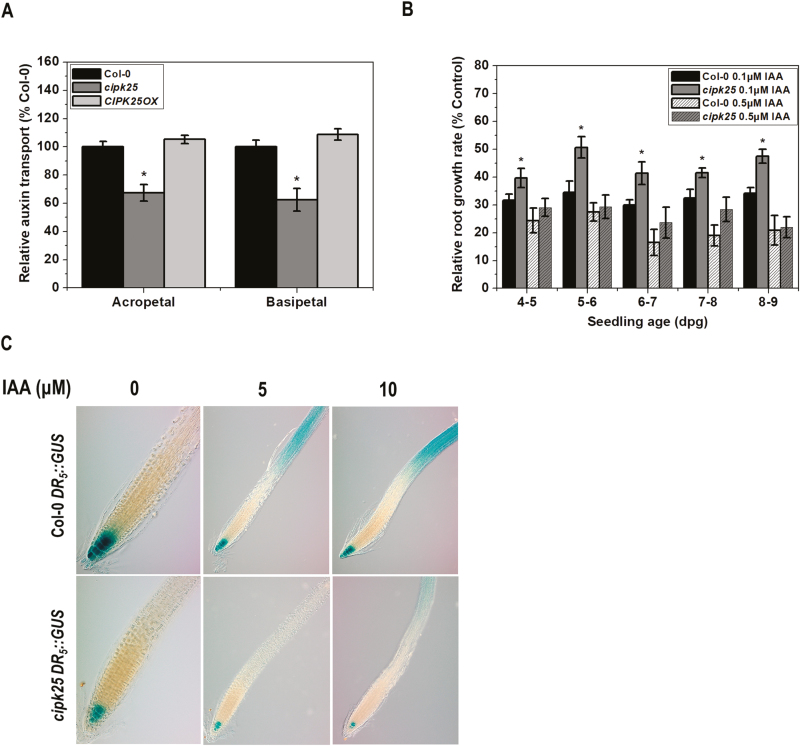
Auxin transport and auxin-responsive promoter assays in *cipk25*. (A) Comparison of acropetal (rootward) and basipetal (shootward) root auxin transport in 5 dpg Col-0, *cipk25*, and *CIPK25OX* seedlings in a root segment 2 mm below (in acopetal) or above (in basipetal) the site of [^3^H]IAA application. Data are presented as the percentage of auxin transport relative to Col-0. Error bars are ±SD, *t*-test (*P≤0.05) of three measurements of 15 seedlings each. (B) Auxin-mediated root growth rate retardation of Col-0 and *cipk25* in response to 0.1 µM and 0.5 µM auxin treatments. Col-0 and *cipk25* mutant seedlings grown on 1/2 MS medium were transferred at 4 dpg to IAA-free or IAA-containing medium, and grown for an additional 5 d. Relative root growth rate in the presence of external IAA is presented as a percentage of the growth rate (per day) of the same line in control conditions (without IAA). The error bars represent ±SD, *t*-test (*P≤0.05), with three replicates of 10 seedlings each. (C) Auxin-responsive promoter activity using *DR*_*5*_*::GUS* in Col-0 and the *cipk25* mutant. Seedlings at 5 dpg were treated with 5 µM and 10 µM IAA for 2 h and stained for GUS activity.

Cell division and patterning in the root are tightly controlled by the local concentration of auxin, which is regulated by a process commonly referred to as polar auxin transport. Acropetal (rootward) and basipetal (shootward) modes of auxin transport in root were assayed using radiolabeled IAA. The mutant line exhibited reduced acropetal and basipetal auxin transport by ~32% and ~38%, respectively, in comparison with Col-0 plants, while the *CIPK25OX* line displayed similar auxin transport to that in Col-0 by both modes (Fig. 5A).

External treatment with auxin is known to retard root growth (Eliasson *et al.*, 1989). To ascertain the effect of external auxin, 4-day-old seedlings of *cipk25* and Col-0 were transferred to media containing 0.1 µM and 0.5 µM IAA, and incubated for 5 d. Exogenous application of 0.1 µM IAA caused relative root growth rate inhibition in Col-0 seedlings by 65–70%. In contrast, relative root growth rate inhibition of *cipk25* seedlings was 48–60%, suggesting that the mutant was less responsive to auxin-mediated root growth retardation, and a higher concentration of auxin was required for a similar effect to that in Col-0. With higher concentration (0.5 µM) of IAA, the difference in relative root growth rate retardation between Col-0 and *cipk25* was not significant (Fig. 5B).

Auxin-responsive gene expression in roots was assessed by the activity of the auxin-responsive promoter–reporter *DR*_*5*_*::GUS*. Col-0 plants with *DR*_*5*_*::GUS* were crossed with *cipk25* to develop a homozygous mutant line expressing *DR*_*5*_*::GUS* (*cipk25 DR*_*5*_*::GUS*). GUS activity in the RAM of 5-day-old *cipk25* seedlings was much lower than in Col-0, implying reduced auxin activity in the RAM of the mutant line. Further, external application of auxin (5 µM and 10 µM IAA for 2 h) caused weaker induction of the auxin-responsive promoter in the *cipk25* root as compared with that in Col-0, suggesting lower auxin transport/signaling in the mutant line (Fig. 5C). A similar contrasting pattern of *DR*_*5*_*::GUS* activity was observed in the case of the *agr1-5 pin2* mutant, which was impaired in basipetal auxin transport (Shin *et al.*, 2005).

### Reduced expression of auxin-responsive genes including auxin efflux carriers in the *cipk25* root

**Fig. 6.**
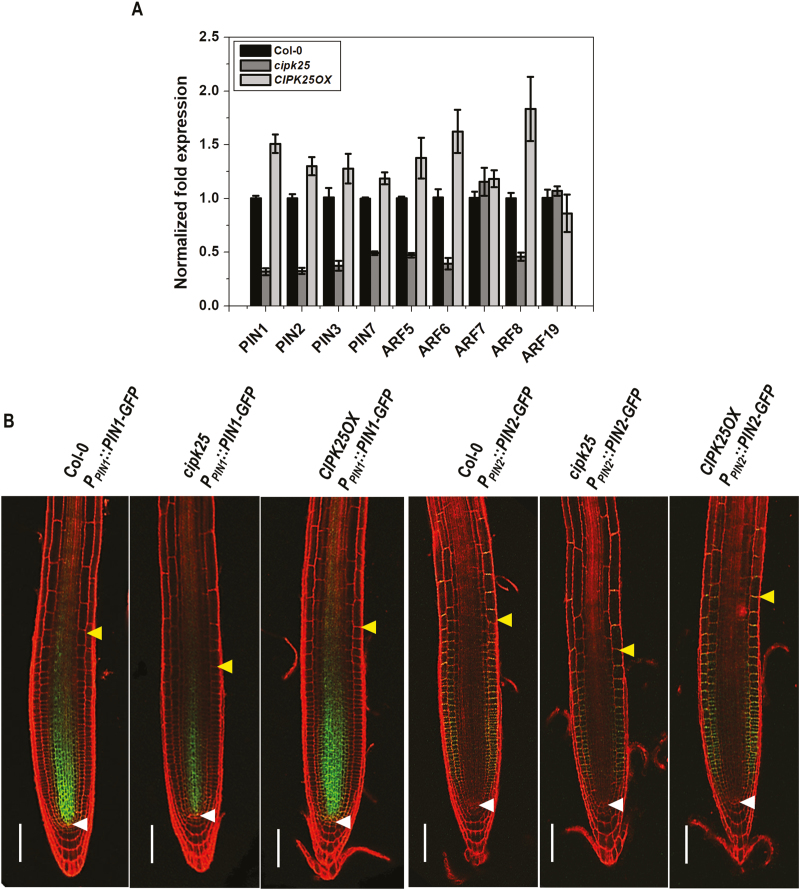
Expression of auxin response factors (ARFs) and efflux carriers (PINs) in *cipk25*. (A) Relative transcript levels of auxin response factors (*ARF5*, *ARF6*, *ARF7*, *ARF8*, and *ARF19*) and efflux carriers (PIN1, PIN2, PIN3, and PIN7) were analyzed by qRT-PCR in roots of 5 dpg Col-0, *cipk25*, and *CIPK25OX* seedlings. *ACTIN2* expression was used for normalization. Error bars represent ±SD of three biological replicates with 15 seedlings for each genotype. (B) Visualization of expression of auxin efflux carriers PIN1 and PIN2. Fluorescence images from the GFP fusion proteins expressed through the native promoters of individual genes in the Col-0, *cipk25*, and *CIPK25OX* background. Seedlings at 5 dpg were stained with propidium iodide to explore meristem cell profiles and analyzed by confocal microscopy. Scale bars in confocal images=100 µm. Lower and upper arrows indicate the quiescent center (QC) and the ﬁrst elongated cortex cell, respectively.

The PIN auxin efflux carriers control growth and patterning in Arabidopsis root (Blilou *et al.*, 2005). ARFs, such as ARF6 and ARF8, were shown to be the positive regulators of adventitious root initiation (Gutierrez *et al.*, 2012). MONOPTEROS/ARF5 is required for primary root formation and vascular development (Berleth and Jürgens, 1993; Przemeck *et al.*, 1996). ARF7 and ARF19 were shown to regulate lateral root formation (Okushima *et al.*, 2007). Expression levels of PINs and ARFs were analyzed by qRT-PCR in roots of Col-0, *cipk25*, and *CIPK25OX* plants (Fig. 6A). *PIN1*, *PIN2*, and *PIN3* expression levels were down-regulated by ~3-fold in *cipk25* seedlings, while an ~2-fold reduction was observed for *PIN7*. Out of five ARFs, expression levels of *ARF5*, *ARF6*, and *ARF8* were down-regulated by >2-fold in *cipk25* as compared with wild-type plants, whereas expression levels of *ARF7* and *ARF19*, involved in lateral root formation, did not show a significant change in *cipk25*. *ARF* and *PIN* genes, which showed reduced expression in the *cipk25* line, exhibited ~1.5- to 2-fold higher expression levels in the roots of the *CIPK25OX* line. Auxin is known to be transported through the central root tissue towards the tip and then redistributed to the cortical tissue and transported basipetally through the epidermis (Swarup and Bennett, 2003; Petrásek and Friml, 2009). The PIN protein auxin efflux carriers facilitate this polar auxin transport. PIN1 is thought to promote auxin movement towards the root tip (acropetal) (Blilou *et al.*, 2005), whereas basipetal auxin transport is dependent on PIN2 (Müller *et al.*, 1998; Abas *et al.*, 2006). To examine the expression of PIN proteins in the absence of CIPK25 expression, *cipk25* mutant lines were crossed with reporter lines expressing PIN1 and PIN2 proteins fused with GFP under their native promoters (*P*_*PIN1*_::*PIN1-GFP* and *P*_*PIN2*_::*PIN2-GFP*). Fluorescence imaging of roots of 5-day-old seedlings showed that expression of PIN1 protein was considerably reduced in the stele of the root in the *cipk25* background. Similarly, the absence of *CIPK25* caused a significant reduction in PIN2 protein expression in the epidermis. Expression of GFP-fused PIN1 and PIN2 was higher in *CIPK25OX* plants (Fig. 6B). All these results suggested that CIPK25 was required for optimal expression of some members of auxin efflux carriers and ARFs.

### SHY2 loss of function rescued the short root phenotype of *cipk25*

**Fig. 7.**
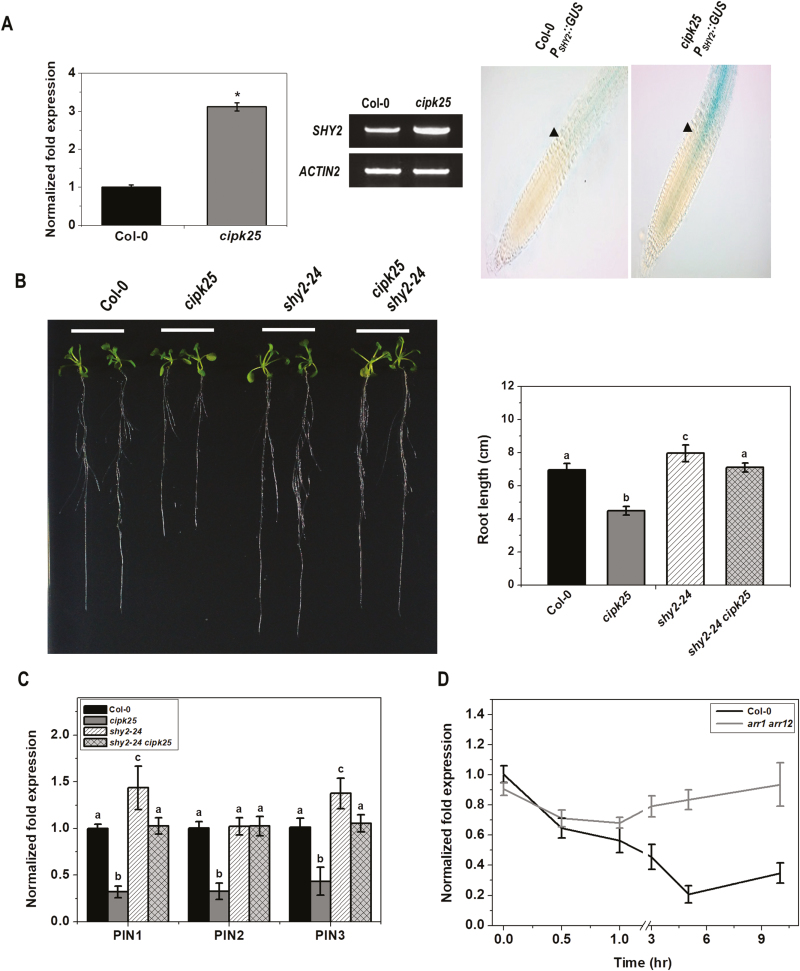
Genetic interaction of *CIPK25* and *SHY2*. (A) Relative transcript abundance of *SHY2* in 5 dpg Col-0 and *cipk25* mutant roots was analyzd by qRT-PCR and semi-quantitative RT-PCR. *ACTIN2* was used as the internal control. The right-hand panel shows histochemical GUS staining of primary roots of 5 dpg seedlings expressing the GUS reporter under the *SHY2* promoter. The arrow shows the first elongated cortex cell. (B) Primary root length phenotype of 10 dpg Col-0, *cipk25*, *shy2-24*, and *cipk25 shy2-24* mutants grown vertically on 1/2 MS medium. The right-hand panel shows root length measurement. Error bars represent ±SD, *n*=24. (C) Relative transcript levels of auxin efflux carriers were analyzed by qRT-PCR in roots of 5 dpg Col-0, *cipk25*, *shy2-24*, and *cipk25 shy2-24* seedlings. Different letters indicate statistically signiﬁcant differences by ANOVA, *P*≤0.05. Error bars are ±SD, *n*=3. (D) qRT-PCR analysis of the expression levels of *CIPK25* in roots of 10 dpg Col-0 and *arr1 arr12* seedlings treated with 5 µM *t*-zeatin for different time intervals. *ACTIN2* expression was used as the internal control. Error bars are ±SD, *n*=3.

The number of cells in the root meristem is regulated by auxin and cytokinin, which promote cell division and cell elongation, respectively. SHY2/IAA3 was proposed to act as a point of intersection of auxin- and cytokinin-mediated signaling to regulate root meristem size. Cytokinin represses *PIN1*, *PIN3*, and *PIN7* expression to influence root meristem maintenance in a SHY2-dependent manner (Tian *et al.*, 2002; Blilou *et al.*, 2005; Vieten *et al.*, 2005; Dello Ioio *et al.*, 2008). As CIPK25 was found to influence root meristem size and PIN expression, the *SHY2* expression level was investigated in *cipk25* and was found to be ~3-fold higher than in the roots of wild-type seedlings. This observation was substantiated by *in planta* expression of the *SHY2* promoter–GUS fusion construct (*P*_*SHY2*_*::GUS*). The *GUS* reporter gene was cloned under a 2.6 kb long *SHY2* promoter and was expressed in wild-type Arabidopsis. It was crossed with the *cipk25* line to generate the *cipk25 P*_*SHY2*_*::GUS* line. The *SHY2* promoter showed a very weak activity in the TD and upwards in the wild-type background, whereas a high *SHY2* promoter activity was observed in the *cipk25* background (Fig. 7A).

We explored the influence of SHY2 on *CIPK25* expression by assessing it in the SHY2 loss-of-function mutant *shy2-24*. In *shy2-24*, a stop codon was introduced at amino acid position 61, which caused truncation of the protein, and the mutant was considered as a null mutant for *SHY2* (Tian and Reed, 1999). *CIPK25* expression in *shy2-24* was equivalent to that in Col-0 (Supplementary Fig. S2). To investigate the genetic interaction between CIPK25 and SHY2, a *cipk25 shy2-24* double mutant was generated. The short root phenotype of the *cipk25* plants was completely recovered in the *cipk25 shy2-24* mutant, resulting in a similar root length to that of the wild-type plants (Fig. 7B). *PIN1*, *PIN2*, and *PIN3* expression levels in *cipk25 shy2-24* were found to be equivalent to those of the wild-type plants (Fig. 7C), indicating that CIPK25 is genetically epistatic to SHY2. Cytokinin treatment down-regulated expression of *CIPK25* (Fig. 4D). Cytokinin control of root meristem size is mediated through ARR1 and ARR12 expression in the TD (Dello Ioio *et al.*, 2007, 2008). Absence or overexpression of *CIPK25* did not cause appreciable changes in *ARR1* and *ARR12* expression (Supplementary Fig. S3). Moreover, cytokinin treatment did not change *CIPK25* expression in the *arr1 arr12* background, suggesting that cytokinin-mediated down-regulation of *CIPK25* expression was dependent on ARR1/ARR12 (Fig. 7D). An analysis of the *CIPK25* promoter using PlantPan2.0 (Chow *et al.*, 2015) showed a number of potential ARR1- and ARR12-binding sites (Supplementary Table S2). All these observations suggested an involvement of CIPK25 in cytokinin signaling mediated through ARR1/ARR12.

**Fig. 8.**
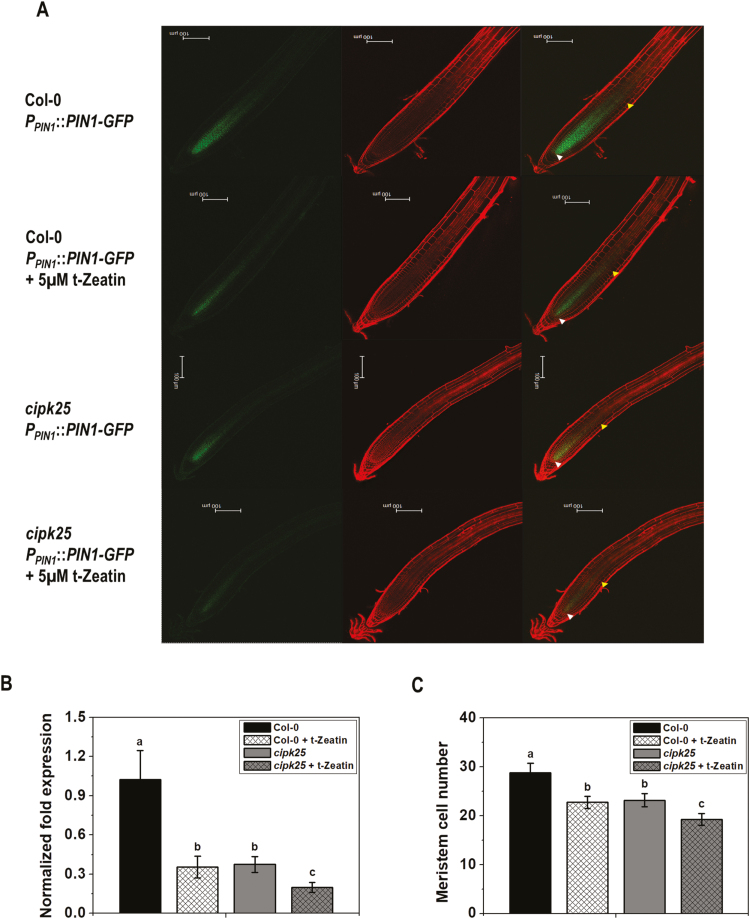
**C**ytokinin-responsive PIN1 expression in *cipk25*. (A) Fluorescence images of auxin efflux carrier PIN1–GFP fusion protein driven by its native promoter in Col-0 and *cipk25* seedlings in control conditions and in response to cytokinin treatment (5 μM *t*-zeatin, 6 h). Roots of 5 dpg seedlings were stained with propidium iodide (PI) for visualizing root cells. Lower and upper arrows indicate the quiescent center (QC) and the ﬁrst elongated cortex cell, respectively. Scale bar=100 µm. (B) Relative transcript levels of *PIN1* were analyzed by qRT-PCR in roots of 5 dpg Col-0 and *cipk25* seedlings treated or not with cytokinin (5 μM *t*-zeatin, 6 h). Error bars are ±SD of three biological replicates, ANOVA, *P*≤0.05. (C) Meristem cell number count in 5 dpg Col-0 and *cipk25* seedlings in control conditions and in response to cytokinin treatment (5 μM *t*-zeatin, 12 h). Values are the mean ±SD of 20 samples each. Different letters indicate signiﬁcant differences among samples (ANOVA; *P*≤0.05).

Cytokinin-mediated down-regulation of PIN1 expression in roots was shown to be dependent on SHY2 (Dello Ioio *et al.*, 2008). To determine whether down-regulation of PIN1 expression by cytokinin is completely dependent on CIPK25, wild-type (Col-0) and *cipk25* plants expressing *P*_*PIN1*_::*PIN1-GFP* were treated with 5 μM *t*-zeatin. Cytokinin treatment resulted in marked down-regulation of PIN1 protein expression in the wild-type background. A reduction in PIN1 expression in *cipk25* roots in control conditions was already shown earlier. Treatment with cytokinin further reduced PIN1 expression in the *cipk25* root (Fig. 8A). This observation was substantiated by *PIN1* gene expression in the *cipk25* root. Cytokinin treatment resulted in an ~3-fold reduction in *PIN1* expression in the wild-type root. A similar (~3-fold lower) *PIN1* expression level was observed in control *cipk25*. Cytokinin treatment caused further reduction in the *PIN1* expression level in *cipk25* by 1.8-fold (Fig. 8B). Additional reduction in meristematic cell number in *cipk25* after cytokinin treatment corroborated reduced PIN1 expression (Fig. 8C). This result suggested that cytokinin regulates root meristem size by both CIPK25-dependent and -independent pathways.

## Discussion

Post-embryonic root growth is maintained by the RAM. Highly proliferative cells of the RAM move longitudinally towards the shoot and eventually differentiate. Hence, cell division and differentiation rates must be equal in order to maintain root meristem size and ultimately overall root development. It has been proposed that a balance of auxin and cytokinin signaling regulates the transition of dividing cells to differentiated cells by activating expression of *SHY2* at the TD, which subsequently decreases expression of PIN genes (Dello Ioio *et al.*, 2008) by suppressing auxin signaling and defines the position of the TD by reducing auxin transport and, additionally, by auxin degradation (Di Mambro *et al.*, 2017). It was reported that *SHY2* expression in the root was minimal at 3 dpg in an actively growing meristem and reached its highest level at 5 dpg, when the meristem attained its final size (Moubayidin *et al.*, 2010). By the same analogy, *SHY2* expression must also be restricted to maintain an equal rate of cell division and rate of transition to elongation. Our results suggest that CIPK25 negatively regulates *SHY2* expression to maintain root meristem size. *CIPK25* expression initiated at the TD and was intense in the differentiated zone, and its expression level was highest when *SHY2* attained its highest expression level after the establishment of the meristem. *SHY2* expression is induced by cytokinin treatment in an ARR1-dependent manner (Moubayidin *et al.*, 2010), while cytokinin treatment decreased *CIPK25* expression in an ARR1/ARR12-dependent manner. A high *SHY2* expression was observed in the absence of CIPK25. All of these observations suggested an antagonistic relationship between SHY2 and CIPK25. However, application of cytokinin caused a further reduction in PIN1 expression and meristem size in the *cipk25* mutant, suggesting that cytokinin also functions in CIPK25-independent pathways. The CIPK25-dependent pathway seems to be totally dependent on SHY2, as a SHY2 loss-of-function mutant complemented the short root phenotype of *cipk25* and restored expression of *PIN1*, *PIN2*, and *PIN3*. Therefore, CIPK25 may be considered as one of the candidate molecules that balance activities of auxin and cytokinin during root development.

As reported before (Schmid *et al.*, 2005), *CIPK25* displayed the highest expression in flowers (Fig. 4). Interestingly, like *ARF6* and *ARF8*, *CIPK25* was also expressed in the stamen and gynoecium, and its expression was greatly reduced in *arf6 arf8* flowers (Reeves *et al.*, 2012). *ARF6* and *ARF8* showed reduced expression in *cipk25* roots. ARF6 and ARF8 are required for flower maturity and growth of the ovule integument (Nagpal *et al.*, 2005; Wu *et al.*, 2006). CIPK25 also displayed a high expression in ovule integuments. We did not observe any abnormal phenotype in the flowers of *cipk25* plants. This might be due to the fact that expression of *ARF6* and *ARF8* was only reduced, but not abolished, in *cipk25*.

CBL4 and CBL5 interacted with CIPK25 in yeast two-hybrid and *in planta* BiFC assays. It is known from several previous reports that an individual CBL protein can interact with multiple CIPKs and vice versa, depending on context (Luan, 2009). It is believed that this flexibility of interaction and their subcellular localizations are crucial in sensing and responding to specific signals. CBL4 was previously shown to interact with CIPK24 and CIPK6 in the context of salinity stress to modulate the activity of the Na^+^/H^+^ antiporter (Qiu *et al.*, 2002) and plasma membrane targeting of the potassium channel AKT2 (Held *et al.*, 2011). Both CBL4 and CBL5 possess conserved domains for myristoylation and acylation to localize in cell membranes (Batistič *et al.*, 2010; Saito *et al.*, 2018). Arabidopsis CIPK25 protein, although it possesses an MG-motif, does not undergo *N*-myristoylation (Saito *et al.*, 2018). It is possible that a major pool of the expressed Arabidopsis CIPK25 protein interacted with the corresponding CBL(s) of *N. benthamiana* and was targeted to the plasma membrane. CBL sequences possess evolutionarily conserved motifs (Mohanta *et al.*, 2015). Cross-species interaction of CIPKs and CBLs was reported before (Tripathi *et al.*, 2009; Wang *et al.*, 2012). Plasma membrane localization might be essential for CIPK25 function. CIPK11 interacts with CBL5, and myristoylation and acylation of CBL5 were shown to be required for the CBL5–CIPK11 complex to phosphorylate the guard cell anion channel SLAC1 (Saito *et al.*, 2018).

 Auxin and cytokinin together regulate various aspects of a plant’s growth and development. The balance between their interactions is maintained by negative regulation of one signaling pathway by the other. On the other hand, they regulate each other’s biosynthesis (Nordström *et al.*, 2004; Jones *et al.*, 2010). The cytokinin biosynthetic gene ATP/ADP isopentyl transferase 5 (IPT5) is rapidly induced by auxin and further induced in the SHY2 loss-of-function background, whereas auxin-dependent induction of *P*_*IPT5*_*::GUS* was totally abolished in the *SHY2* gain-of-function background (Dello Ioio *et al.*, 2008). Relative enhancement of cytokinin activity at the root transition domain might reduce *CIPK25* expression, leading to increased *SHY2* expression resulting in reduced auxin signaling. We propose CIPK25 functions genetically above SHY2. Cytokinin-mediated suppression of *CIPK25* expression was dependent on ARR1/ARR12. ARR1 is able to regulate *SHY2* expression directly by binding to and activating the *SHY2* promoter (Dello Ioio *et al.*, 2008). It is noteworthy that cytokinin was able to suppress PIN1 expression even in the absence of CIPK25, suggesting a partial role for CIPK25 in balancing auxin–cytokinin signaling in root meristem development. Presently, it is not clear how CIPK25 functions in auxin/cytokinin signaling. Auxin and cytokinin are involved in many interconnected developmental processes through various cross-connected signaling pathways. Therefore, a role for CIPK25 in other pathways related to root development cannot be ruled out.

It is known that extracellular application of calcium inhibits cell growth in the TD (Ishikawa and Evans, 1992; Legué *et al.*, 1997; Fasano *et al.*, 2002). Ca^2+^ plays a vital role in polarity and rate of auxin transport through PIN proteins by modulating endo- and exocytosis and the direction of PIN localization in the plasma membrane (reviewed in Vanneste and Friml, 2013). In this study, we proposed that a Ca^2+^-regulated protein kinase, CIPK25, is involved in regulation of root meristem size by regulating auxin transport through modulating PIN protein expression.

## Supplementary data

Supplementary data are available at *JXB* online.

Table S1. Primers used in this study.

Table S2. A list of auxin- and cytokinin-responsive elements in the CIPK25 promoter.

Fig. S1. Detection of YFP^N^–CIPK25, CBL4–YFP^C^, and CBL9–YFP^C^ fusion proteins in BiFC assay.

Fig. S2. qRT-PCR analysis of the expression levels of *CIPK25* in the *shy2-24* line.

Fig. S3. Relative transcript abundance of *ARR1* and *ARR12* in *cipk25* and *CIPK25OX* seedlings.

Supplementary Figures S1-S3

Supplementary Tables S1-S2
