# Supplementary material for: CBL-interacting protein kinase 25 contributes to root meristem development
Source: J Exp Bot. 2018 Sep 18;70(1):133–47. doi: 10.1093/jxb/ery334 (PMC6305191; doi:10.1093/jxb/ery334)
Supplement: Supplementary Figures S1-S3 [file ery334_suppl_supplementary_figures_s1-s3.pdf]

Supplementary Figures:

CBL-interacting protein kinase 25 contributes to root meristem development

Mukesh Kumar Meena, Niraj Kumar Vishwakarma, Vineeta Tripathi, Debasis Chattopadhyay*

National Institute of Plant Genome Research, Aruna Asaf Ali Marg, New Delhi 110067, India
To whom correspondence should be addressed. Email: debasis@nipgr.ac.in

This file includes:

Supplemental Figure S1. Detection of YFP^N-CIPK25, CBL4-YFP^C and CBL9-YFP^C fusion proteins in BiFC assay.

Supplemental Figure S2. qRT-PCR analysis of the expression levels of *CIPK25* in *shy2-24* line.

Supplemental Figure S3. Relative transcript abundance of *ARR1* and *ARR12* in *cipk25* and *CIPK25OX* seedlings.

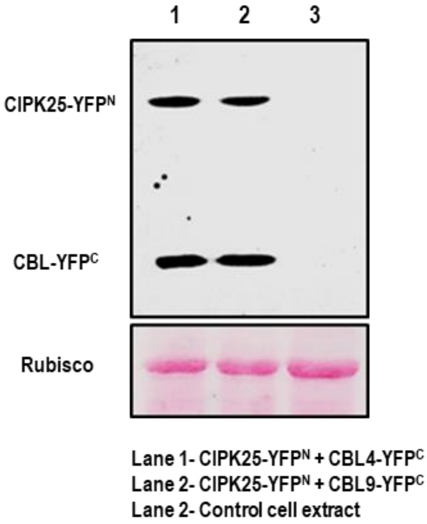

Fig. S1. Detection of YFP^{N}-CIPK25, CBL4-YFP^{C} and CBL9-YFP^{C} fusion proteins in BIFC assay with polyclonal anti-GFP antibody. *Agrobacterium tumefaciens* (GV3101) infilterated tissue was loaded as control. Rubisco detected by Ponceau S staining of the blot.

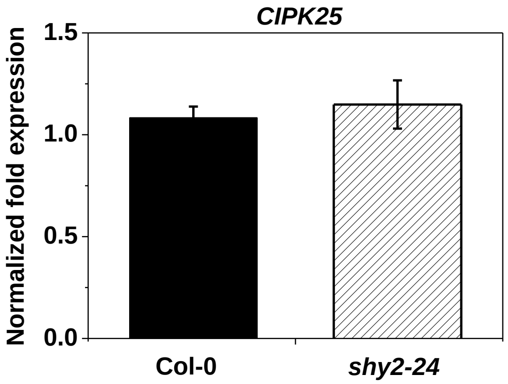

Fig. S2. Normalized fold expression of *CIPK25* in roots of 5 day-old Col-0 and *shy2-24* seedlings as determined by qRT-PCR. *ACTIN2* expression was used as internal control. Values are mean SD± of four biological replicates with 15 seedlings each.

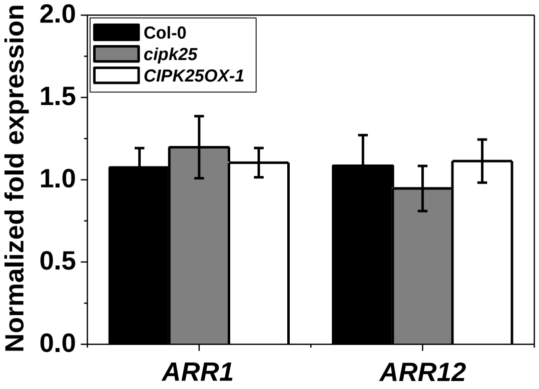

Fig. S3. Normalized fold expression of Arabidopsis type B cytokinin response regulators *ARR1* and *ARR12* were analyzed by qRT-PCR in roots of 5 day-old Col-0, *cipk25* and *CIPK25OX-1* seedlings. *ACTIN2* expression was used for normalization. Values are mean SD ± of four biological replicates with 15 seedlings each.
