# Supplementary material for: CBL-interacting protein kinase 25 contributes to root meristem development
Source: J Exp Bot. 2018 Sep 18;70(1):133–47. doi: 10.1093/jxb/ery334 (PMC6305191; doi:10.1093/jxb/ery334)
Supplement: Supplementary Tables S1-S2 [file ery334_suppl_supplementary_tables_s1-s2.pdf]

Supplementary Tables:

CBL-interacting protein kinase 25 contributes to root meristem development

Mukesh Kumar Meena, Niraj Kumar Vishwakarma, Vineeta Tripathi, Debasis Chattopadhyay

National Institute of Plant Genome Research, Aruna Asaf Ali Marg, New Delhi 110067,India

To whom correspondence should be addressed. Email: debasis@nipgr.ac.in

Table S1: Primers used in this study.

Table S2. A list of auxin- and cytokinin- responsive elements in CIPK25 (AT5G25110.1) promoter sequence

Table S1: Primers used in this study.

	**Primer**	**Sequence**
1	LBb1	5'-GCGTGGACCGCTTGCTGCAACT-3’
2	*cipk25-1* LP	5’-AACAAATTCATCATCATGGCG-3’
3	*cipk25-1* RP	5’-TCCAACAATTAATCTCAGCCG-3’
4	*cipk25-2* LP	5’- TTGGATAATTGGTTTGGATCG -3’
5	*cipk25-2* RP	5’- CGAAGTTCTTTAACGCGTTTG -3’
6	PRMCIPK25/FP	5’-CGGGATCCGGCCTAGAGGTATCTAACTT-3’
7	PRMCIPK25/RP	5’-CGGGATCCCATGTGTATATACAGAAGTA-3’
8	CIPK254T2F	5’-TCCCCCGGGATGGGATCCAAACTTAAACT-3’
9	CIPK254T2R	5’-TCCCCCGGGTTAGCAGTCACTACCAGAAT
10	CIP25SDM/L	5’-GGTTTGCTTCATGATCAGTGTGGAACT-3’
11	CIP25SDM/R	5’-AGTTCCACACTGATCATGAAGCAAACC-3’
12	CIPK25CFY	5'-GGAATTCCATATGGCGATTATGCGAACACCGTG-3'
13	CIPK25RY	5'-GGAATTCCATATGTTAGCAGTCACTACCAGAAT-3'
14	CIPK25FY	5'-GGAATTCCATATGGGATCCAAACTTAAACT-3'
15	CBL1F	5’-GGAATTCCATATGGGCTGCTTCCACTCAAA-3'
16	CBL1R	5'-CGGAATTCTCATGTGGCAATCTCATCGA-3'
17	CBL2F	5'-GGAATTCCATATGTCGCAGTGCGTTGACGG-3'
18	CBL2R	5'-CGGAATTCTCAGGTATCTTCAACCTGAG-3'
19	CBL3F	5'-GGAATTCCATATGTCGCAGTGCATAGACGG-3'
20	CBL3R	5'-CGGAATTCTCAGGTATCTTCCACCTGCG-3'
21	CBL4F	5'-GGAATTCCATATGGGCTGCTCTGTATCGAA-3'
22	CBL4R	5'-CGGAATTCTTAGGAAGATACGTTTTGCA-3'
23	CBL5F	5'-GGAATTCCATATGGGATGTGTTTGCAGCAA-3'
24	CBL5R	5'-CGGAATTCTTACCGGAGAAAGGTTGGGA-3'
25	CBL6F	5'-GGAATTCCATATGATGATGCAATGTTTAGA-3'
26	CBL6R	5'-CGGAATTCTCATCCATCCAGCTCACTAG-3'
27	CBL7F	5'-GGAATTCCATATGGATTCAACAAGAAATTC-3'
28	CBL7R	5'-CGGAATTCTCAGGTATCTTCCACTTGCG-3'
29	CBL8F	5'-GGAATTCCATATGTTGGCATTCGTGAAATG-3'
30	CBL8R	5'-CGGAATTCCTAGTCTTCAACTTCAGAGT-3’
31	CBL9F	5'-GGAATTCCATATGGGTTGTTTCCATTCCAC-3'
32	CBL9R	5'-CGGAATTCTCACGTCGCAATCTCGTCCA-3'
33	CBL10F	5’-CGGGATCCATATGGAACAAGTTTCCTCT-3’
34	CBL10R	5'-CGAGCTCGTCAGTCTTCAACCTCAGTGT-3'

35	AtCIPK25prom 2.6k FP	5’-CGGGATCCTATCAAAAGTCTGGGTCAAAC-3’
36	AtCIPK25prom 2.6k RP	5’-CGGGATCCGTGTATATACAGAAGTAGAATG-3’
37	AtSHY2 prom 2.6k FP	5’-ACGCGTCGACGTGGTGGTTAGATTGAAGTG-3’
38	AtSHY2 prom 2.6kRP	5’- GCTCTAGACCCTTCTTCAAGAATTGCAGGAG-3’
39	CAMCIPK25/L	5’- CCCATGGGGATGGGATCCAAACTTAAACT-3’
40	CAMCIPK25/R	5’- GACTAGTTTAGCAGTCACTACCAGAAT-3’
41	Act2 F RT	5’-TCAGATGCCCAGAAGTCTTGTTC-3’
42	Act2 R RT	5’-GTGGATTCCAGCAGCTTCCA-3’
43	AtCIPK25 F RT	5’-CCAGCGTTGGAGGAGATAGTGT-3’
44	AtCIPK25 R RT	5’-CAGTCACTACCAGAATTTTCATCACTAA-3’
45	ARR1 RT F	5’-TGCTAACCTCTTCGCAATCATC-3’
46	ARR1 RT R	5’-TCTCGGAAATGCGGTTTGA-3’
47	SHY2 RT N F	5’-CGGGAACAGATAATGTATGTGAA-3’
48	SHY2 RT N R	5’-TGTTGTTATTACAGCAAGAAACTCTC-3’
49	PIN1 RT F	5’-CACTTCATCGCCGCTAACAA-3’
50	PIN1 RT R	5’-GAATCTGCGGCGAGGAAAC-3’
51	PIN2 RT F	5’-CCGTGGGGCTAAGCTTCTCATCT-3’
52	PIN2 RT R	5’- AGCTTTCCGTCGTCTCCTATCTCC-3’
53	PIN3 RT F	5’-GAAAATCTTCTCACCCGACCAA-3’
54	PIN3 RT R	5’-GGCGAAGATGGCGACAAA-3’
55	PIN7 RT F	5’-CGGCATAAACCGCTTCGT-3’
56	PIN7 RT R	5’-TTGAGGAGATGAAGTGGAAAGAGA-3’
57	ARF5 RT F	5’-GCCACGTAGAGCTGCAGAGAA-3’
58	ARF5 RT R	5’-TGGCGGCTGTGCTGAGT-3’
59	ARF6 RT F	5’-CGCGCCATCTTCCAACA-3’
60	ARF6 RT R	5’-CGTCACAGTTCCCGTTGCT-3’
61	ARF7 RT F	5’-TGACGGTGATTCCAGGAACA-3’
62	ARF7 RT R	5’-AGGCACAAAGCCATTATCAACA-3’
63	ARF8 RT F	5’-GTGCATCAAATGGGAGATCATG-3’
64	ARF8 RT R	5’-TCGGGTTTTGCGGGAAT-3’
65	ARF19 RT F	5’-TGGAGGACTGTGGCCCAAT-3’
66	ARF19 RT R	5’-ACCCTCGTTTTTGAACCTTTGTAT-3’
67	AtCIPK25prom SmaI FP	5’-TCCCCCGGGTATCAAAAGTCTGGGTCAAAC-3’
68	AtCIPK25 SmaI RP	5’- TCCCCCGGGTTAGCAGTCACTACCAGAAT-3’
69	AtCIPK25F-attB1	5’-GGGGACAAGTTTGTACAAAAAAGCAGGCTTCATGGG ATCCAAACTTAAACTTTAC-3’
70	AtCIPK25F-attB2	5’-GGGGACCACTTTGTACAAGAAAGCTGGGTTGCAGTC ACTACCAGAATTTTCATCAC-3’

Table S2. A list of auxin- and cytokinin- responsive elements in CIPK25 (AT5G25110.1) promoter sequence

-2116

AATGTGTAATTAGTGAACTTGAAACTTTTGAAAAATAATTTGAAATATCTCGTTA
TACGATTAATTATATGAAAATTCATAGAAAATGTGATGCTAATAGGAATATAGCA
ATTTGGGGGAAAAATCTAATGAAAAAGGATTTTATGAAACTTCATATATTATTAC
TATTTTTTGTTAAACATGAAATTTGGAGTGACTTCAAACAAATTAAAAATATGAA
ATCTCCCATATATACAACATCAACGTAAAAAAACAAAAAAAAAGCCTCATTCAT
GCCTCGTTATCGTCGGATTCTATCGTATTGATCTCTTCATGGTAGTCTCCATTATT
CATCTTGACCATTCGTTGTTCTCATGTCTCTCTTAATCTCCGGTTAAGCTCTGATT
CTCACTTTGATCTCTTGTTCGATCTTTCTACTATTATTATTGCATTTACTATTTAAA
ATTTTATATCCTAGTAGTTGGATCTCTAATCTCTTATTAACAAATTATTGACGTAG
ATGACAAAAAAATAAAAAAAATCATTGACGTTAATCTTACACATTATGTTCTGTG
AGGTAGTAGAACAATTTTGGAGGTAACACTTTTCTTTCACAGTTGTTGTCTTCGTC
AAAACTATGGGCCTAGAGGTATCTAACTTTTGATACGAAAATTCTCCTTAGCAGC
ATTTTAAAAAAATCATAAGAGCCCACTGAATCTCAAGCCTTTTTATAATCATTAG
AATATATTTGATATTTATTAAAAGCCCACTAATTTTAAAATCCACCAAAATAATC
AAAATCCTTAATCCAATAGACCCTTAAATAGTTAAATTATTTAGAAACTCTAACA
AATTCTTTTATTTAACAACCTTCCTAAGCTTAAAGTTTGTAATTGTTTTGTATTTTT
CCCAAAGTTTTAGTATCTTGATATTTTAAGATCGTTCTATAAATAGATTTGATTTT
GTAAGTTGTTTATATTGTTACTAGGCCTTTGTATAAATACCAAATACTTTGATTAT
TTTTTTACTGATTTCATGTCAAACAAAATTCCAAACAACTTAAAGGAAAAAGCTT
ACTCGATTGGAATAAAAATTGTACGTTTAACAAAATGATATTAGTGAAATAAATA
AACCAGCTCAACATTTAAGGATTTCTACGTGTATATACAGTAATCGTGTTTAGTA
TTAATTAGCTATAGTTTAGCCTCAAAAAACAAAGATCCAAAACAACATGACGAA
ATTGACTTCCACATCGTGTCCTTTCTCGTCGACATAAAAACTTTAATATATAGATG
CATGTATAAGTATACTCACTATTTGTGTCATTAGTATTTTCTTTTTTGATACTTGG
AGCTAATGACAAAAATTCCGCTAGAAATATCTTCTCCAACGATAGCTACAAACA
AATACTACCATTGCGTCATCATCACTTATTTATATTCGATATTTCAGTCAACAACA
CTAATCTCTTTCACCAAAATCTTTGGACTATTATTACAAATGAATTAAAAACTTG
GACCCTTAATAACATATTTGTTTCATTAAATCTATACAACTCTTATTAAAAATGAT
ATTTTCTTAAATCAAAAATTAATCAAAATTAAGGTAGGCTCTAGAGGATTGCATC
ATTTGCCCACCCACATTAGCCTGCCATTATTATTGGACGGTAACAAATTTCCTTAC
ACCAATAAATGGTAAGTTACAATTATTATTAACATTTCTATATAAAATAGTTTGG
TTATTTACATTAATTTTTATTTAATACTATAAACTTAAAAATTCAATGCTGAAAAG
TGTAACGGAAAGAGGGACATTAAATATATGTTAATATAATAAGTATATTTACTAT
TTAAAAAAGACTTATGTAAATACATATTGTATAGAGAGAAAAATAACCCATAGT
ATAAAAACTTGCATCTCTAAAACCGCAGCATTGATAATAAGAGTCTCTTTCTTCT
TAAGTATCTCTTAATTAACCCACATAATATATTTACAAGTGGCCGCCTCATACAT
CCCCTCTCAAGTCCTTTGCTTCATTTCTTATAAAACTAAACCACCTTCTTCTTCAT
CATTCATCACCAATCTATCTCATATTCCATATACTGAATATGGACTCATTCTACTT
CTGTATATACAC

TFs	Position	Strand	Sequence	Similar Score	
ARF1	-1646	+	TGTCTc	1	**Auxin Responsive**
ARF2	-1646	+	TGTCTc	1	
ARF9	-1646	+	TGTCTc	1	
ARF10	-1646	+	TGTCTc	1	
ARF11	-1646	+	TGTCTc	1	
ARF12	-1646	+	TGTCTc	1	
ARF13	-1646	+	TGTCTc	1	
ARF16	-1646	+	TGTCTc	1	
ARF17	-1646	+	TGTCTc	1	
ARF18	-1646	+	TGTCTc	1	
ARF21	-1646	+	TGTCTc	1	
ARF23	-1646	+	TGTCTc	1	
ARR1	-1311	+	actGAATCtc	0.981	**Cytokinin Responsive**
ARR2	-1103	+	tagtATCTTg	0.974	
ARR2	-806	-	aAAGATccaa	0.953	
ARR12	-1878	-	AATCT	1	
ARR12	-1864	+	GGATT	1	
ARR12	-1781	-	AATCT	1	
ARR12	-1712	+	GGATT	1	
ARR12	-1636	-	AATCT	1	
ARR12	-1530	-	AATCT	1	
ARR12	-1469	-	AATCT	1	
ARR12	-1307	-	AATCT	1	
ARR12	-1242	-	AATCC	1	
ARR12	-1223	-	AATCC	1	
ARR12	-1216	-	AATCC	1	
ARR12	-1069	+	AGATT	1	
ARR12	-873	+	GGATT	1	
ARR12	-558	-	AATCT	1	
ARR12	-543	-	AATCT	1	
ARR12	-477	-	AATCT	1	
ARR12	-404	+	GGATT	1	
ARR12	60	-	AATCT	1	
ARR14	-1311	+	actgAATCTc	0.982	
ARR14	-1070	-	tAGATTtgat	0.978	
ARR18	-1375	+	gaggTATCTaact	0.942	
ARR18	-1104	+	ttagTATCTtgat	0.947	
ARR18	-63	+	taagTATCTctta	0.942	
ARR19	-1878	-	AATCT	1	
ARR19	-1864	+	GGATT	1	
ARR19	-1781	-	AATCT	1	
ARR19	-1712	+	GGATT	1	

ARR19	-1636	-	AATCT	1
ARR19	-1530	-	AATCT	1
ARR19	-1469	-	AATCT	1
ARR19	-1307	-	AATCT	1
ARR19	-1242	-	AATCC	1
ARR19	-1223	-	AATCC	1
ARR19	-1216	-	AATCC	1
ARR19	-1069	+	AGATT	1
ARR19	-873	+	GGATT	1
ARR19	-558	-	AATCT	1
ARR19	-543	-	AATCT	1
ARR19	-477	-	AATCT	1
ARR19	-404	+	GGATT	1
ARR19	60	-	AATCT	1
